# Incidence of bony Bankart lesions in Sweden: a study of 790 cases from the Swedish fracture register

**DOI:** 10.1186/s13018-023-04173-x

**Published:** 2023-09-13

**Authors:** Vladislavs Gordins, Mikael Sansone, Baldur Thorolfsson, Michael Möller, Malin Carling, Nicklas Olsson

**Affiliations:** 1 Institute of Clinical Sciences, Sahlgrenska Academy, University of Gothenburg, 413 45 Gothenburg, Sweden; 2 Department of Orthopaedics, Sahlgrenska University Hospital, Göteborgsvägen 31, 431 80 Mölndal, Sweden

**Keywords:** Bony Bankart, Glenoid rim fracture, Anterior shoulder instability

## Abstract

**Background:**

A bony Bankart lesion directly affects the stability of the shoulder by reducing the glenoid joint-contact area. The aim of this study was to report on the epidemiological data relating to bony Bankart lesions in Sweden using the Swedish fracture register. The purpose is to evaluate age and sex distribution in the population with bony Bankart lesions, its impact on treatment strategy and further to analyse patient-reported outcomes.

**Methods:**

This was an epidemiological descriptive study. The inclusion criteria were all patients with a unilateral bony Bankart lesion registered between April 2012 and April 2019. The patients’ specific data (age, sex, type and time of injury, treatment option and patient-reported outcomes) were extracted from the Swedish fracture register database.

**Results:**

A total of 790 unilateral bony Bankart fractures were identified. The majority of the patients were male (58.7%). The median age for all patients at the time of injury was 57 years. Females had a higher median age of 66 years, compared with males, 51 years. Most of the bony Bankart lesions, 662 (91.8%), were registered as a low-energy trauma. More than two-thirds of all treatment registered cases, 509/734 patients (69.3%), were treated non-surgically, 225 (30.7%) were treated surgically, while, in 17 patients (7.5% of all surgically treated patients), the treatment was changed from non-surgical to surgical due to recurrent instability. Surgical treatment was chosen for 149 (35%) of the males and for 76 (25%) of the females. Patient quality of life decreased slightly in both surgically and non-surgically treated groups 1 year after bony Bankart injury.

**Conclusion:**

This national register-based study provides detailed information on the epidemiology, choice of treatment and patient-reported outcomes in a large cohort of bony Bankart lesions. Most bony Bankart lesions affected males between 40 and 75 years after low-energy falls and non-surgical treatment dominated.

## Background

The glenohumeral joint is one of the most mobile joints in the human body and is, therefore, one of the most unstable as well [1]. The most common type of glenohumeral instability is anterior, accounting for more than 90% of all shoulder dislocations [2, 3]. One of the most common associated injuries, following anterior shoulder dislocation, is the Bankart lesion [4]. This is an injury to the anterior–inferior glenoid labrum with or without a bone fragment, and it is usually associated with an inferior glenohumeral ligament (IGHL) complex injury [4]. A Bankart lesion, which includes a bone fragment, is usually called a bony Bankart [5]. A bony Bankart lesion occurs when the injury to the anterior glenoid labrum extends into the bony glenoid margin, creating a fracture line through the anterior–inferior part of the glenoid, and thereby reduces the area of the glenoid and increases the risk of subsequent instability [6–10]. Bony Bankart fragments can differ in size and form and can directly affect the stability of the shoulder by reducing the joint-contact area and congruency [11–13].

There is ample evidence of isolated soft-tissue Bankart lesions and risk factors predisposing to first-time or recurrent traumatic anterior shoulder dislocations [14]. It has been reported that, in younger age groups, this type of injury is more common in males, especially between 20 and 30 years of age, whereas, after the age of 50, it is considered more common among women [2, 15, 16]. The risk of recurrence after an anterior shoulder dislocation is high, particularly in younger patients, and can lead to osteoarthritis (OA) later in life [15]. Young patients with no concurrent fracture at the time of the primary shoulder dislocation have been shown to have a high risk of recurrence [16]. Moreover, the recurrence rate is highest in individuals aged ≤ 20 years, where nearly 50% will require surgical stabilisation [17].

There is a lack of consensus, and only limited data in the literature related to the treatment strategy for a bony Bankart injury. It is known that surgical treatment for large bony Bankart fragments (more than 20% of the glenoid width) plays a crucial role in improving patients’ clinical outcomes [18], and it has been shown to be a successful treatment strategy for athletes [19]. On the other hand, it has also been shown in some studies that even large and displaced glenoid rim fractures can be treated non-surgically [20, 21], especially when the glenohumeral joint is concentrically reduced [20, 22].

As a result, there is a need to collect more data in order to produce evidence relating to the most used treatment strategy for and epidemiology of bony Bankart lesions. High-quality epidemiological data will increase the understanding of this injury. This knowledge can be used for further studies related to fracture types in relation to treatment options.

The aim of this study is to report epidemiological data relating to bony Bankart lesions in Sweden using the Swedish fracture register. The purpose is to evaluate age and sex distribution in the population with bony Bankart lesions and its impact on treatment strategy and further to analyse patient-reported outcomes.

## Materials and methods

Data from the Swedish fracture register (SFR) were extracted. The SFR was developed in 2009–2010 [23]. Data registration started in 2011 at the Sahlgrenska University Hospital. Since 2012, more departments in Sweden have been invited to join, and, in 2021, full national coverage was achieved among the 54 orthopaedic and trauma departments in the country. More than 300,000 fractures were registered in the SFR [24] by 2018.

**Fig. 1:**
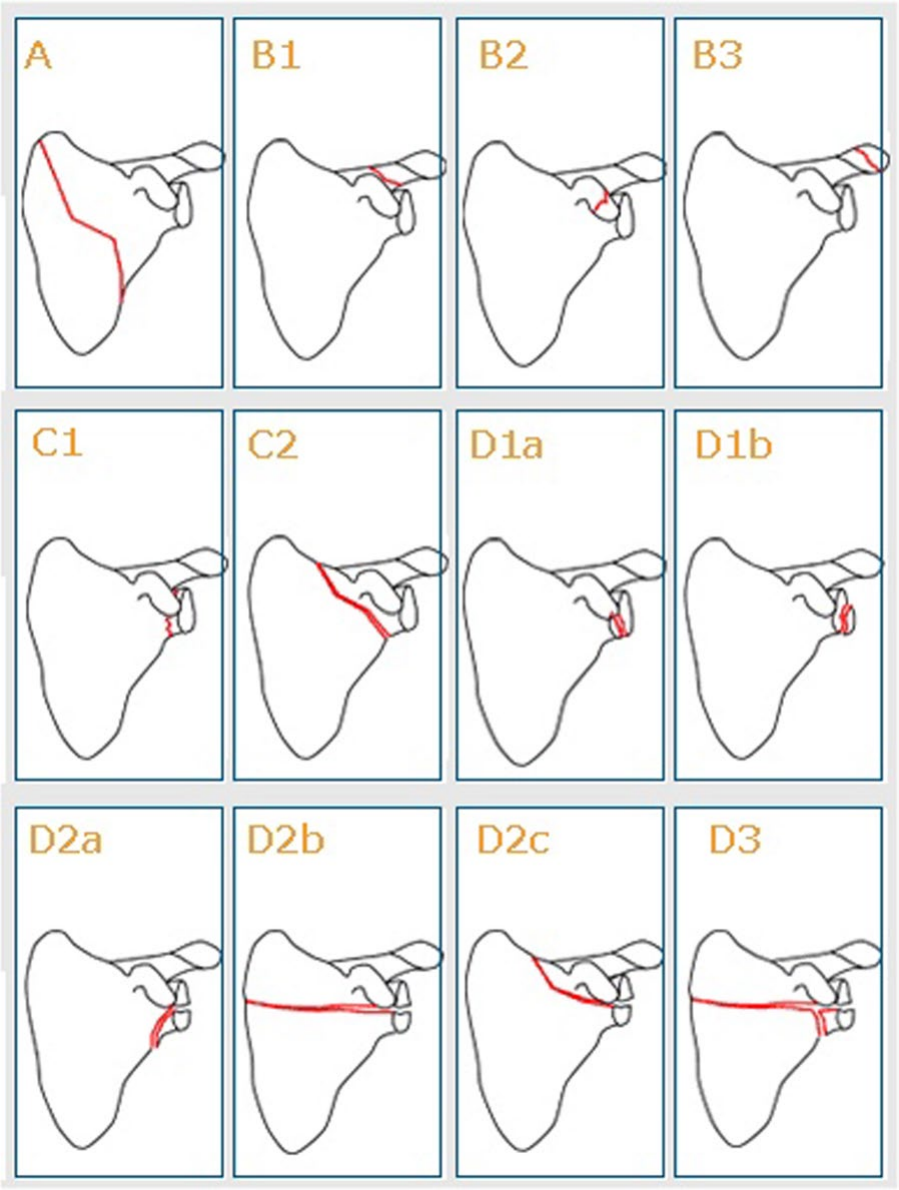
Modified Euler and Rüedi classification of scapular fractures

In the SFR, the modified Euler and Rüedi classification, edited by Habermeyer in 1996, is used to classify different types of glenoid fracture [25]. Data are reported to the register by the treating physician at the emergency orthopaedic department. We defined a bony Bankart injury as all patients with a registered type D1a injury according to the modified Euler and Rüedi classification (Fig. 1).

Data for these patients from 1 April 2012 to 1 April 2019 in the SFR were extracted.

Epidemiological data in terms of sex, age, cause of injury and type of treatment were collected from the SFR and analysed.

Injuries were classified based on trauma mechanism, as high- or low-energy trauma, based on the criteria from the ATLS guidelines [26]. A low-energy trauma is usually associated with injury mechanisms such as falling from standing height or less.

The type of treatment was divided into three groups: non-surgical, primary surgical or secondary surgical (when a primary decision to perform non-surgical treatment had been revised at an early stage).

The EQVAS score was used to evaluate patient-related outcomes [27]. This score ranges from 0 to 100, with 0 denoting the worst and 100 the best possible health state imaginable. The first measurement referred to as PROM0 presents patient quality of life before the injury, and the second measurement, PROM1, presents it 1 year after the injury. The first PROM was obtained using the recall technique during the 1st weeks after the fracture occurred.

The study was approved by Ethical Committee confirming that all methods were carried out in accordance with relevant guidelines and regulations (Dnr: 825-18).

### Statistics

Categorical variable numbers (*n*) are presented in per cent. For continuous variables, the mean ± standard deviation (SD)/median value (range) in numbers is presented, depending on whether or not data were normally distributed. For comparisons between groups, Fisher’s exact test (lowest one-sided *p* value multiplied by 2) was used for dichotomous variables.

If no exact limits could be computed, the asymptotic Wald confidence limits with continuity correction were calculated instead. For comparisons within groups, the Wilcoxon signed-rank test was used. The IBM SPSS Statistics, Version 2, statistical software (SPSS Inc., Chicago, USA) was used.

## Results

We identified 790 patients with unilateral bony Bankart injuries. Only one patient with a bilateral bony Bankart injury was reported and was not included in this study.

### Epidemiology

**Fig. 2:**
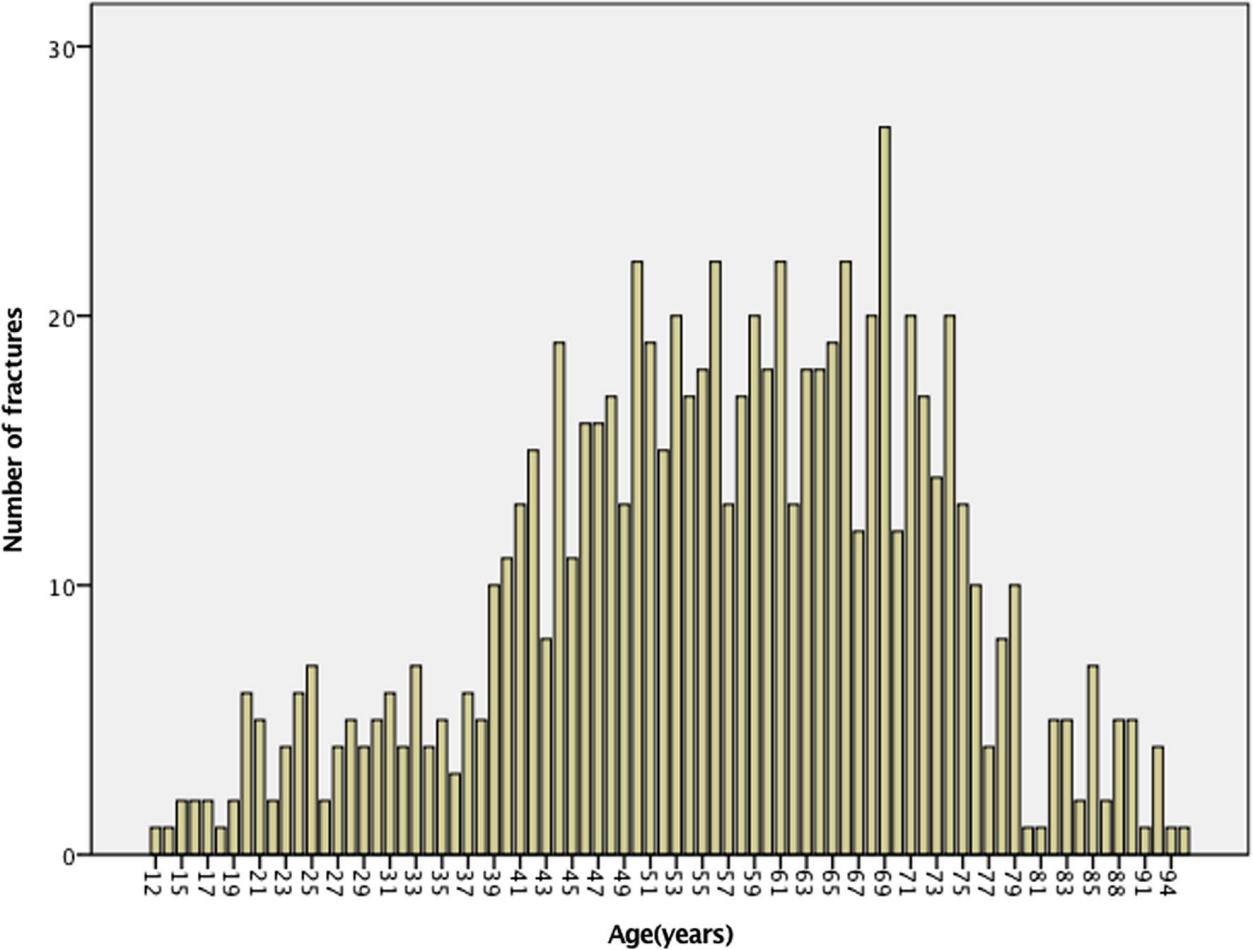
Distribution of BB injury per age interval

Of 790 patients, 58.7% were male (Table 1). The median age for all patients at the time of injury was 57 years (Fig. 2). Females had a higher median age of 66 years, compared with males, 51 years.

**Table 1:** Median age and sex at the time of injury

Variable	*n* = 790
Age^a^	
Median (min–max)	57 (12–95)
Gender, *n* (%)	
Female	326 (41.3)
Male	464 (58.7)

^a^Age at the time of injury

In 721 (91.2%) of the patients, the trauma energy level was defined. Most of the bony Bankart lesions; 91.8% (662 of 721), were registered as a low-energy trauma.

A fall from the same level was the most common cause of injury or 494 (62.5%), while a fall from a height occurred in 83 (10.5%) of the patients. Traffic accidents accounted for 41 (5.2%) of all injuries.

Bony Bankart lesions occurred in the right shoulder in 404 (51.1%) and the left shoulder in 386 (48.9%) patients.

**Fig.3:**
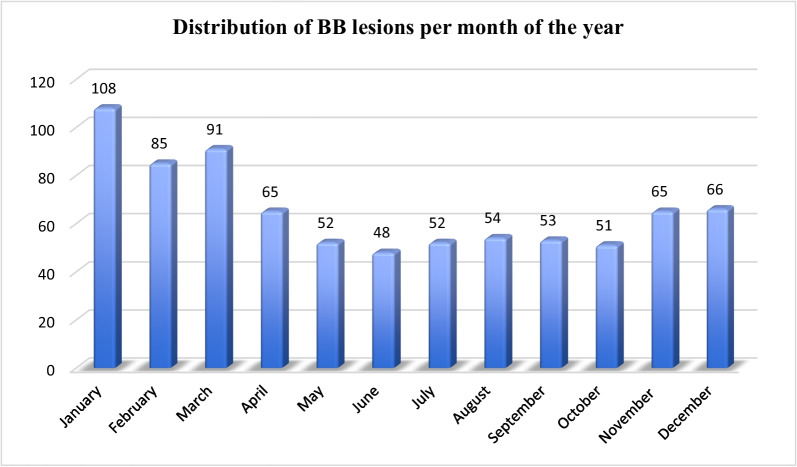
Distribution of BB lesions per month

For the distribution of injuries over the year, please see Fig. 3. Thirty-five per cent of the injuries occurred during the first quarter of the year.

### Treatment strategy

**Fig. 4:**
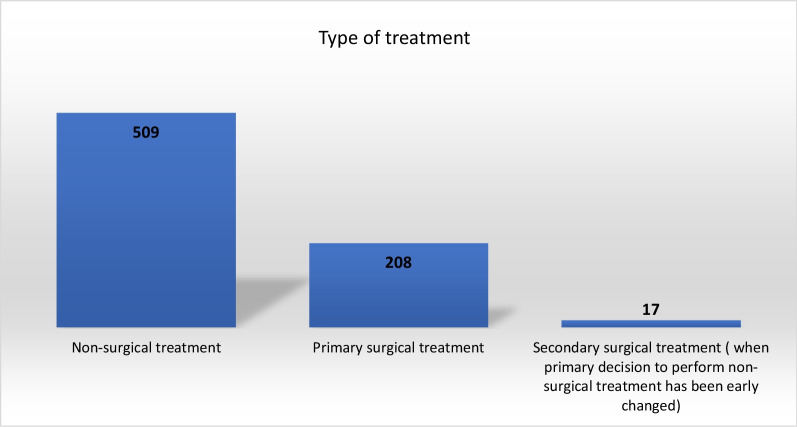
Type of treatment for the BB lesions (missing—56)

More than two-thirds of all cases with registered treatment data (missing value in 56 patients), 509/734 patients (69.3%), were treated non-surgically; 225 (30.7%) were treated surgically. In 208, surgical treatment was primarily chosen and secondary in 17 patients (7.5%) when the treatment was changed from non-surgical to surgical, due to recurrent instability of the shoulder (Fig. 4). The median age of the patients in the surgical group was similar to that of the non-surgical group (55 vs. 58 years) (Table 2).

**Table 2:** Median age of the patients for different treatment options (missing—56)

Variable	Non-surgical treatment *n* = 509	Primary surgical treatment *n* = 208	Secondary surgical treatment *n* = 17
Age^a^			
Median (min; max)	58 (15; 95)	55 (19; 83)	56 (21; 79)

^a^Age at the time of injury

Surgical treatment was chosen for 149 (35%) of males and 76 (25%) of females (Table 3).

**Table 3:** Treatment type distribution in relation to sex

Variable	Men (*n* = 428)	Women (*n* = 306)
Treatment type		
Non-surgical treatment	279 (65.2%)	230 (75.2%)
Primary surgical treatment	140 (32.7%)	68 (22.2%)
Secondary surgical treatment	9 (2.1%)	8 (2.6%)
Missing	36	20

**Fig. 5:**
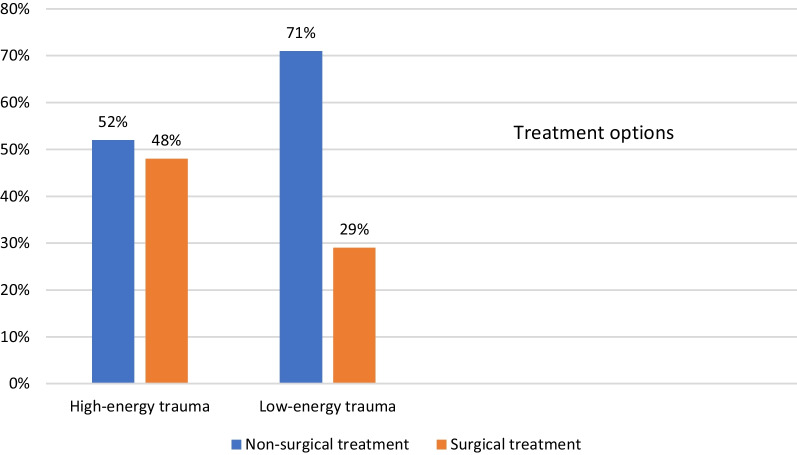
Treatment options for high- and low-energy-induced BB injuries

Bony Bankart lesions induced by high-energy trauma were more often treated surgically (Table 4 and Fig. 5), and 26 (48%) of the patients in the high-energy trauma group were treated surgically, compared with 175 (29%) in the low-energy trauma group.

**Table 4:** Choice of treatment for high- and low-energy-induced bony Bankart lesions

Variable	Non-surgical treatment (*n* = 459)	Surgical treatment (*n* = 201)	*p* value	Difference between groups mean (95% CI)
Type of injury				
High energy	28 (6.1%)	26 (12.9%)		− 6.8 (− 12.3; − 1.3)
Low energy	431 (93.9%)	175 (87.1%)	0.0067	6.8 (1.3; 12.3)
Missing	130	130

Patient quality of life decreased slightly in both the surgically and non-surgically treated groups 1 year after the bony Bankart injury (Table 5).

**Table 5:** Quality of life changes in the non-surgically and surgically treated groups

Variable	Median (min; max) *n* =	*p* value within group
*Non-surgically treated group (n = 509)*		
EQVAS (prom0)	90.0 (0.0; 100.0) *n* = 185	
EQVAS (prom1)	80.0 (0.0; 100.0) *n* = 100	
EQVAS difference between pro0 and pro1	− 5.00 (− 80.00; 80.00) *n* = 88	< 0.0001
*Surgically treated group (n = 225)*		
EQVAS (prom0)	90.0 (1.0; 100.0) *n* = 84	
EQVAS (prom1)	85.0 (40.0; 100.0) *n* = 47	
EQVAS difference between prom0 and prom1	− 5.00 (− 50.00; 57.00) *n* = 44	0.031

## Discussion

The most important finding of this study is the reporting of many epidemiological variables of this injury. This was made possible by the large data sample in the SFR.

The completeness of the SFR differs among fracture types, and there are no data that report the completeness of bony Bankart fractures, type D1a. Möller et al. have shown completeness of approximately 85% for hip and femur fractures and 70% for wrist fractures [28].

The present study shows that bony Bankart lesions are more frequent in the population over 50 years of age. This is contrary to primary anterior shoulder dislocation which usually occurs in the young population with the median age of 35 years [29]. One reason why a bony Bankart lesion is more typical in older patients could be biomechanical and biological changes leading to reduced elasticity of the joint capsule and possibly weaker bone quality at higher age [30, 31].

The prevalence of male gender in all types of primary anterior shoulder dislocation is around 70% [29, 32]. The present study shows minor predominance for bony Bankart lesions in males, 58.7%.

A simple, low-energy fall was one of the most common causes of this injury. This study further reveals a seasonal variation in the distribution of bony Bankart lesions. This type of injury is more common during the winter period, possibly related to slippery conditions.

Most bony Bankart lesions were treated non-surgically, particularly in females. This could be related to fracture size, the position of the fracture related to differences in ligament laxity, bone quality or other factors [31, 33]. The age of the patients did not affect the treatment strategy.

A higher prevalence of non-surgical treatment could be related to the relatively limited degree of dislocation of the bony Bankart fragment, a low complication rate and good bone-to-bone healing potential [34, 35]. Olds et al. reported a low risk of recurrent instability in the presence of a bony Bankart lesion [14]. Robinson et al. reported an increased risk of recurrence in the presence of a glenoid rim fracture during the first 6 weeks following a first-time traumatic anterior shoulder dislocation in only 3.2% of patients [36].

One-third of bony Bankart lesions; 225/734 (30.7%), were treated surgically, which is similar to previously published data on the treatment of soft-tissue Bankart lesions [37, 38]. In the present study, we found that the majority of the bony Bankart lesions were due to low-energy trauma and were mostly treated non-surgically (71% of all patients), compared with high-energy bony Bankart lesions, which were treated surgically to a greater extent. Almost half of the patients (48%) with bony Bankart lesions induced by high-energy trauma were treated surgically, which could be related to more dislocated and larger bony Bankart fragments due to high-energy forces.

It is difficult to draw any definitive conclusion in terms of patient quality of life 1 year after the injury due to the limited response rate. However, quality of life appears to decrease slightly in both groups, especially in the non-surgically treated group. These results were statistically significant, albeit not clinically relevant, since the minimal clinically important change on the EQVAS has been shown to supersede the attained values [39].

Moreover, we compared the data with previously published epidemiological studies based on the SFR analysing other types of fractures [40–42]. Some differences and common features were found. Most fractures had a similar seasonal variation with an increased number of injuries during the winter months and a higher incidence after the age of 40 years. The main difference between studies was the distribution of gender. The majority of bony Bankart lesions occurred in males and decreased dramatically after the age of 75 years compared with other fractures [40–42].

These results may suggest that bony Bankart injuries are probably not related to fragility or reduced bone mineral density.

There are several limitations to the present study. One of them was the low response rate in PROMS, especially at the 1-year follow-up. Another limitation is that the modified Euler and Rüedi classification could lead to misclassification, causing difficulties for surgeons to classify the fracture in some non-standard cases. The simplicity of this classification that has been used to classify different types of glenoid fracture in the SFR is an advantage, compared with the AO (Arbeitsgemeinschaft für Osteosynthesefragen) classification of scapular fractures from 2013. However, it is incomplete and can make it difficult to distinguish isolated anterior glenoid fractures with a large anterior fragment from bony Bankart lesions in several patients [43].

One of the main strengths of this study is the large amount of data from the Swedish fracture register, including non-surgically treated fractures. The current register-based study provides reliable data on bony Bankart epidemiology in Sweden.

## Conclusion

This national register-based study provides detailed information on the epidemiology, choice of treatment and patient-reported outcomes in a large cohort of bony Bankart lesions. Most bony Bankart lesions affected males between 40 and 75 years of age after low-energy falls and were treated non-surgically.

## Data Availability

The datasets used and analysed during the current study are available from the corresponding author on reasonable request.

## Abbreviations

IGHL: Inferior glenohumeral ligament
OA: Osteoarthritis
SFR: Swedish fracture register
